# Internet addiction test: Croatian preliminary study

**DOI:** 10.1186/s12888-019-2366-2

**Published:** 2019-12-05

**Authors:** Iva Černja, Lucija Vejmelka, Miroslav Rajter

**Affiliations:** 1 University Department for Croatian Studies, University of Zagreb, Borongajska cesta 83d, 10000 Zagreb, Croatia; 2 Department of Social Work, Faculty of Law, University of Zagreb, Nazorova 51, 10000 Zagreb, Croatia; 3 Research Office, University of Zagreb, Ul. kralja Zvonimira 8, 10000 Zagreb, Croatia

**Keywords:** Internet addiction test, Adolescents, Gender differences, Croatian sample

## Abstract

**Background:**

Everyday internet usage is particularly significant in the population of adolescents and young people. Besides numerous benefits, internet usage brings certain risks of addictive behavior. Internet Addiction Test (IAT) is the most spread scale for measuring internet addiction. The aim of this study was to investigate internet addiction on a sample of Croatian adolescents.

**Methods:**

Overall, 352 students aged between 15 and 20 from randomly selected high schools participated in the study. We have collected the data on the Internet Addiction Test along with basic demographic information. The main analyses included the factorization of IAT test and the measurement of prevalence of internet addiction with the analysis of the gender differences.

**Results:**

The results showed that 3.4% of high school students reported high levels of internet addiction, while 35.4% of respondents reported some signs of addiction. Three-factor structure of IAT was obtained with dimensions: *Emotional and cognitive internet preoccupation*, then *Neglecting work and lack of self-control* and the last one is *Social problems*. Although the first factor has the most significant role in internet addiction risk, gender differences were found only in the last two factors, where boys have higher scores on *Social Problems*, while girls have higher scores on *Neglecting work and lack of self-control*.

**Conclusions:**

Based on our results, the prevention activities should be focused on the area of ​​emotional and social competence and the responsible use of internet. Since the result show that a third of the sample show moderate signs of addiction, programs of both indicated and selective prevention should be systematically planned for the general population of adolescents as well as for the groups in risk. The obtained gender differences indicate that the preventive and treatment programs should take into account gender specifics.

## Background

Everyday use of the internet provides a number of advantages in professional and private activities, but the virtual environment also provides settings for new types of risk behaviors of individuals. The problematic use of the internet includes the phenomenon of internet addiction. The internet addiction has been in a focus of researchers and experts over the last two decades, and Dr. Kimberly Young is the author of the Internet Addiction Test tool that has been widely used for research and detection purposes of addictive symptoms in individuals [53]. The aforementioned author also carried out the first empirical study on internet addiction in 1996, and she has been systematically investigating this field since then.

Although internet addiction is not included in the official classification system of mental illness and disorder, and there are no formal diagnostic criteria available in the International Classification of Diseases and Related Health Problems (ICD-10) (WHO, [51]) and DSM-5 [2], except online gaming disorder. On Ansembly of WHO in May 2018 ICD 11 was presented for adoption by Member States and with this changes Internet gaming disorder has been classified as psychiatric disorder in the International Classification of Diseases and Related Health Problems (ICD-11). Implementation of ICD 11 will start at 1 January 2022 allowing the states to adapt and prepare the changes in their national policies [52]. On the other hand, the phenomenon of internet addiction is recognized by practitioners and professionals. Internet addiction centers have been active in the treatment of addictive behavior for a number of years ([37], http://netaddiction.com/). Mihajlov and Vejmelka [35] state that internet addiction treatment has recently been present in the practice of Croatian medical institutions and that today there are departments of daily hospitals for the treatment of addiction on the internet as well as individual and group treatment at certain psychiatric departments (http://www.pbsvi.hr). Although the addiction treatment practice on the internet is beginning to develop in some countries, there is a lack of research in this area.

In literature, it is possible to find different terminology for the same phenomenon such as “compulsive internet use”, “excessive use of the internet”, “problematic use of the internet”, “pathological internet usage”, “internet addiction”, “computer dependence” and “net addiction” ([39]., [21]., [55].). In this paper, we will use the term internet addiction. Young [53] describes internet addiction as an impulse control disorder that does not include narcotics, while the extended definition of the same author states that this is a condition in which an individual loses control over the use of the internet and continues to use it excessively to the point of experiencing problematic outcomes with negative influences [54]. The difficulties encountered by people who use the internet excessively are categorized into five areas: academic, interpersonal relationships, financial, professional and physical [53].

### Internet addiction test

Young [53] has developed an instrument for diagnosing the problematic internet use called Internet Addiction Diagnostic Questionnaire (IADQ), which lists eight addiction criteria [53]:

- the person is preoccupied with the internet,
- there is a need to spend more and more time on the internet to achieve satisfaction;
- unsuccessfully attempts to control, reduce, or interrupt the use of the internet;
- feels anxiety and depression in reducing or stopping the use of the internet;
- remains on the internet much longer than it is intended;
- endangers personal contacts, job, study, career;
- conceals the truth about addiction from family members and helping professional;
- uses the internet to escape the problem.

Based on the IADQ, the most commonly used version of the questionnaire for the measurement of the internet addiction called Internet Addiction Test (IAT) was developed. IAT is a 20-item scale that measures the presence and severity of internet addiction. This test was designed as a research and diagnostic tool, based on the DSM-IV criterion for pathological gambling diagnosis. The IAT is a symptom-measuring tool for the internet addiction. The internet addiction is defined here as online compulsive behavior that causes hindering normal social interactions, and also increases daily stress and feelings of solitude, anxiety, and depression. The test measures the degree of involvement in online activities using responses on the 5-degree Likert type scale and categorizes the addictive behavior into four categories: lack of addiction, mild signs of addiction, moderate signs of addiction, and severe addictive behavior [54]. Studies have confirmed that IAT is a reliable measure covering the key characteristics of pathological internet use. The questionnaire showed good reliability in different countries and with different populations. ([4, 11, 17, 46].

### Research and prevalence of internet addiction in adolescents

Kimberly Young [53] states that between 5 and 10% of internet users are estimated to be addicted to the internet, and two decades of research in this area confirms the spread of this phenomenon on the adolescent population. The internet is used by all age groups, but it is extremely popular among children and young people, where it becomes an inevitable source of information, communication and entertainment in the consumption of free time [48]. Statistically, youth groups account for the largest share of the total number of internet users (from 16 to 24 years [18]. Similar is in Croatia, so the data from the Croatian Bureau of Statistics [14] confirm that the largest share of internet users are young people and that the number of users falls proportionally to their age. Further, the same source states that as many as 96% of young people in the age group of 16 to 34 use the internet [14]. The studies that used internet Addiction Test (IAT) on adolescents show the prevalence rates range from the smallest 0.2% to the maximum 10.9% of the participants that we can classify as an addicted to the internet. Given that this is a relatively new area, there are still different approaches to the definition of this phenomenon among scientists and researchers, different criteria for determining addiction are present, and the specificity of the condition for conducting research and the choice of different methods of conducting research makes it impossible to compare the results of the research.

According to IAT results, in a recent study conducted in Mexico on a sample of 522 persons aged 15–61, 91.8% of respondents use the internet without any sign of addiction, 8% are at risk of addiction, showing moderate signs of addiction while 0.2% of participants in the highly addicted category [7].

In Italy, the results on the sample of 2533 young people aged 14 to 21 show that most do not show any sign of addiction (94%), 5% of young people showed mild to moderate addiction, while only 0.79% of them showed a high level of addiction [38]. In a similar survey in the same country a few years later on a sample of 1035 young people between the ages of 13 and 22, the results were quite different and categorized by 26% of the respondents in the group without the risk of addiction, 70% in the slightly addicted group and 4% in the group of addicted internet users [6].

Authors Cheng and Li [11] published a meta-analysis of 164 independent samples (*N* = 89,281) where the results for 31 countries were presented. The results of their studies indicate that the highest prevalence of high addiction on the internet is found in the Middle East (10.9%). Following are Southeast European countries with a prevalence of 6.1%, while the North and West Europe countries show the lowest percentage of prevalence of internet addiction (2.6%) [11].

Durkee et al. [17] conducted a study on 12,000 adolescents from 11 countries in Europe, according to which the prevalence of high addiction on the internet averages 4.4%, while Tsitsika et al. [47] survey on a sample of 13,248 European adolescents categorizes 1.2% of adolescents in a group of highly addicted.

Although many studies have investigated gender differences in internet usage and internet addiction, there is no clear consensus on who has a higher risk of developing internet addiction [8]. Studies mainly suggest that men achieve higher results in internet addiction [7, 9, 12, 20, 34, 41] although Leung [33] identifies higher level of IA (Internet addiction) among women.). On the other hand, some studies suggest there is no difference in the level of internet addiction between men and women [8], so the further examination of this relationship is needed.

Findings from longitudinal studies are important for better understanding internet addiction phenomena. Longitudinal study of a Stavropoulos et al. [44] examined the role of conscientiousness (as a personality trait) and classroom hostility (as a contextual factor) in the development of IA and interesting results indicated that the contribution of individual and contextual IA factors may differ across genders and over time.

### Dimensions of internet addiction

IA subtypes refers to work of Kimberly Young and her associates. Based on empirical data, Young et al. (1999 in [35]) identifies five subtypes that comprise of a wide variety of behaviors and impulse control problems.

Some researches contribute to development of theoretical frameworks of Internet addiction. Brand et al. [5] suggest an Interaction of Person-Affect-Cognition-Execution (I-PACE) model of specific Internet use disorders - a theoretical framework for the processes underlying the development and maintenance of an addictive use of certain Internet applications or sites promoting gaming, gambling, pornography viewing, shopping, or communication.

Due to the increasing importance of this construct in clinical and research practice, a large number of validation studies of this instrument have been carried out. Validations were conducted in a number of countries and different cultures such as UK [26, 49, 50]; Italy [19]; China [8], France [29], Finland [30], Germany [3], China ([8, 31] [16]., Greece [43], Poland [23], Portugal [39], and Bangladesh [28]. These studies have confirmed the medium to high internal homogeneity of the test, while the research carried out in China and Finland has confirmed good convergent and divergent validity [8, 30]. However, in the results about the factor structure, numerous variations in the structure were determined. For example, Hawi [22] confirm the existence of only one factor, while Korkeila et al. [30] obtained two factors called salience and lack of control. The range of factor structure in these studies is from a single factor to a structure of 6 dimensions. These variations have been determined from study to study, where variations are also observed in countries of similar cultures.

Although the primary focus of this research is not the validation of the instrument, given the large variations in the structure of the test in different countries, it is necessary to establish the dimensions of the IAT for the adequate interpretation of the level of internet addiction on the Croatian sample. The aim of this study is to investigate the level of internet addiction on the individual dimensions of internet addiction and the overall outcome. Given that the data show that the level of internet usage is highest among adolescents, the Internet Addiction Test was applied to a sample of Croatian high school students. With the aim of detecting the prevalence of the internet addiction in this age group, after the categorization of addiction, we will get a clearer insight into the proportion of students with pronounced symptoms of addiction. Given the inconsistent results of sexual differences at the level of addiction, we will examine the level of addiction in particular on the sample of male and female students both on the overall result and on the individual dimensions of IAT. Research findings will contribute to a better understanding of internet addiction in the adolescent population. The research findings have practical implications for planning interventions with children and young people in the field of problematic use of the internet. Given the initial challenges in clinical practice, the results on a sample of young people can be of use to practitioners working with children and young people and provide an additional body of knowledge for the international scientific community.

## Methods

The research was conducted in March and April 2016 in 9 randomly selected secondary schools in Croatia, in 18 classes. The schools from the sample were different sizes from 7 to 44 classes in rural and urban area contributing to representative research results. The study was approved by the Ethics Commission of the Faculty of Law, University of Zagreb, and the implementation of research by school principals. All standards of the Code of Ethics of Conducting Research with Children [1] were applied. According to the section 3 of the national Code of Ethics of Conducting Research with Children for this study, along with the approval from the competent body for the ethical regulation and with the informed verbal consent of the participants, it was necessary to send the written information about the research to their parents prior to the data collection, along with the contact information of the researchers. This procedure was applied in this research providing parents possibility to contact the researchers about the child participation in the research. The parental consent was not collected because under the Ethical code, for children who are over 14 years of age only participants give consent when the research does not involve deception and it does not have potential risks, discomfort, or adverse effects. The participants ware offered the possibility of contacting the relevant organizations who work with children and youth if they wish to discuss topics from the field of research. The data collection was done via web questionnaires that the students filled during their computer science class. Described procedure was approved by Ethical committee of the Faculty of Law at the University of Zagreb.

### Participants

A list of high school institutions [24] was used as a sampling framework for the respondents. The strategy of multistage probabilistic cluster sampling was used, whereby in the first stage, the schools were selected by simple random selection from the list. In the second stage, two classes were selected from each school using random selection with the only criterion that the classes were held in computer cabinets that have the appropriate environment for online survey. In the selected classes, all the students participated in the study.

In the research all the students who were willing to participate in the research from selected classes in each school were enrolled in the study. The average number of students per class was 19.55 while in the same year national average was 22,7 students per class. School miss-outs for different reasons, e.g. health problems and others and possibility not to participate in the study, and there were no refusals for the participation in the study from the students who were present in the classroom. The study involved 352 high school students in Croatia, aged 15–20. The distribution of the pupils by gender and age is shown in Table 1. Of the total number of students, 50 (14.2%) attended a three-year vocational school, 225 (63.9%) four-year vocational school and 77 (21.9%) gymnasium.

**Table 1:** Sample structure by age and gender

	1st grade	2nd grade	3rd grade	4th grade	TOTAL
Boys	83	39	14	59	195
Girls	46	70	25	16	157
TOTAL	129	109	39	75	352
Age - M (sd)	16.0 (.51)	17.0 (.56)	17.80 (.57)	18.73 (.53)	17.06 (1.17)

The random sample of high school students participating in this survey by ratio does not significantly deviate from the sample of the entire population of high school students. In school year 2014/2015 117.384 of students went to vocational schools and 53.652 students went to gymnasiums ([36].). In total 177,661 students were enrolled in high school programs [13].

In the school year 2014/2015. In Croatia, 46.3% of students attended four-year, 21.2% three-year vocational schools, while 32.6% were in grammar schools [36].

Students mainly state that they use internet at home every day (96,3%) while 61.7% of them also use internet in school every day. Children and young people state that they spend 3–4 h a day on the use of multimedia content on the Internet (music and movies) and on social networks and instant messaging.

Girls spend more time on social networks, listening to music, exchanging instant messages, writing homework, searching for medical information, and shopping. On the other hand, boys far more than girls spend time playing online games, on adult content pages, online gambling, chatting, and online forums.

### Instrument

Internet Addiction Test [53] (internet Addiction Test – IAT was applied to measure internet addiction. The test measures the level of addiction on the internet and consists of 20 item with a range of 0 to 5 (e.g. *How often do you stay online longer than you planned? (0 = Never; 1 = Seldom; 2 = Occasionally; 3 = Frequently; 4 = very often, 5 = always*). The total range of the questionnaire is from 0 to 100. Participants can be classified into several categories [50] and the result of 0 to 19 indicates the absence of addiction, from 20 to 39 indicates a low level of addiction and average online user, from 40 to 69 represents a moderate level of addiction, while the result of 70 to 100 assumes severe level of internet addiction. The internal consistency coefficient (Cronbach alpha) on this sample is 0.91.

### Statistical analyses

All of the statistical analyses were conducted using IBM SPSS 23 and R. The factor structure of the IAT was examined using Principal component analysis and verified using Confirmatory factor analysis within the *lavaan* package [40]. The sample was divided into four categories based on the result on IAT and the differences regarding IAT categories and gender on IAT score and the score on IAT factors was tested using MANOVA (Multivariate analysis of variance). Prior to testing the gender differences measurement invariance was tested both on single factor solution and on three factor solution using the measurement Invariance and partial Invariance functions in the *semTools* package [27].

## Results

### Factor structure

The correlation matrix of 20 items of the questionnaire indicates the suitability of the matrix for factor analysis. The value of the Kaiser Meyer Olkin (KMO) coefficient is 0.92 and it is large enough to conclude on the adequacy of the matrix for analysis (Kaiser, 1970). Bartlet’s sphericity test indicates that the extradiagonal elements of the correlation matrix are statistically significantly different from 0 (χ2 = 2743.221, df = 190, *p* < .001). Out of 190 correlations among the items, 188 were statistically significant (*p* < 0.05). All of the item-total correlations were statistically significant (*p* < .05) and ranged from .32 to .75. Only two items have a correlation with a total score of less than .40.

The study used the analysis of principal components with varimax rotation. Initial analysis extracted 3 components with a characteristic root greater than 1, and this structure was supported by the results of the Scree test. Three extracted components explain 51.64% of the total variance. All items on the corresponding components have saturation greater than .40. Component saturation on a single component is shown in Table 2. The first component is called *Emotional and cognitive internet preoccupation - reliance on online life*, the second *Neglecting work and lack of self-control*, and the third *Social problems*.

**Table 2:** Factor structure of IAT items

	IAT items	Factor saturation		
		F1	F2	F3
IAT 20	How often do you feel depressed, moody, or nervous when you are off-line, which goes away once you are back online?	.792		
IAT 15	How often do you feel preoccupied with the internet when off-line, or fantasize about being online?	.739		
IAT 13	How often do you snap, yell, or act annoyed if someone bothers you while you are online?	.690	(.319)	
IAT 18	How often do you try to hide how long you’ve been online?	.648		
IAT 11	How often do you find yourself anticipating when you will go online again?	.630	(.319)	(.337)
IAT 19	How often do you choose to spend more time online over going out with others?	.601		
IAT 12	How often do you fear that life without the internet would be boring, empty, and joyless?	.601		
IAT 14	How often do you lose sleep due to being online?	.499	(.476)	
IAT 10	How often do you block out disturbing thoughts about your life with soothing thoughts of the internet?	.408		(.374)
IAT 8	How often does your job performance or productivity suffer because of the internet?		.780	
IAT 6	How often do your grades or school work suffer because of the amount of time you spend online?		.725	(.301)
IAT 1	How often do you find that you stay online longer than you intended?		.676	
IAT 2	How often do you neglect household chores to spend more time online?		.675	
IAT 16	How often do you find yourself saying “just a few more minutes” when online?	(.517)	.628	
IAT 17	How often do you try to cut down the amount of time you spend online and fail?	(.444)	.600	
IAT 3	How often do you prefer the excitement of the internet to intimacy with your partner?			.627
IAT 4	How often do you form new relationships with fellow online users?			.608
IAT 7	How often do you check your email before something else that you need to do?			.526
IAT 9	How often do you become defensive or secretive when anyone asks you what you do online?	(.311)		.497
IAT 5	How often do others in your life complain to you about the amount of time you spend online?	(.308)	(.397)	.428
Characteristic root		4419	3692	2217
% variance		22,096	18,462	11,084
Cronbach α		0,865	.840	.620

In order to determine the adequacy of the obtained structure, confirmatory factor analysis was performed on three factors. The results indicate that the three-factor model is well-suited to the data (χ^2^/df < 3, RMSEA = .075, CFI = .882, SRMR = .056). Since the values fit indices are within the range of the acceptable values [25, 45] we conclude that confirmatory factor analysis confirmed the obtained structure. Since one of the aims of this paper was to examine gender differences, measurement invariance was tested both on single factor solution and on three factor solution with the maximum likelihood estimation (MLM). Using the criteria of ΔCFI of-0,01 and ΔRMSEA of 0.015 [10] the partial measurement invariance regarding gender was obtained for 18/20 items on complete IAT (exclusions were items “How often do you find yourself anticipating when you will go online again?” and “How often do you try to cut down the amount of time you spend online and fail?“), while on three factor solutions the model achieved full measurement invariance.

### The level of the internet addiction

In order to investigate the level of internet addiction, respondents were divided into four categories. A total of 24.4% of respondents (IAT < 20) show no signs of addiction. Most of the respondents (39%) fit into the low-level addiction category (20 ≤ IAT ≤ 39), 32% of respondents’ show a moderate level of addiction (40 ≤ IAT ≤ 69) and 3.4% of respondents are in the category of severe addiction level (IAT ≥ 70). Given the small number of students with high levels of addiction, one category has been formed which includes a moderate and high level of addiction.

After extraction of three factors, factor scores were formed to investigate the differences in the categories of internet addiction and gender on different components, as well as the overall outcome of the Internet Addiction Test. Descriptive data for total score and factor results are shown in Table 3. It should be noted that the score on factor points is presented as standardized results on z-scale. Table 4 shows the results of MANOVA with gender and addiction risk categories as independent variables and IAT scores by factors as dependent variables.

**Table 3:** Descriptive data of overall score and factor scores by gender and level of addiction risk category

		IAT score		Emotional and Cognitive internet preoccupation		Neglecting Work and Lack of Self-Control		Social Problems	
		M	sd	M	sd	M	sd	M	sd
Male	No addiction (*N* = 55)	10.06	5.425	−.67	.366	−1.02	.460	−.51	.559
	Low level of addiction (*N* = 72)	29.92	5.895	−.18	.674	−.17	.782	.06	.918
	Moderate and severe addiction level (*N* = 68)	52.25	1.918	.69	1.238	.56	.958	.60	1.293
*TOTAL (N = 195)*		32,256	18.362	−.01	1.022	−.15	.996	.10	1.079
Female	No addiction (*N* = 31)	13.45	3.632	−.76	.333	−.64	.497	−.54	.479
	Low level of addiction (*N* = 64)	29.63	5.296	−.24	.731	.02	.760	−.18	.870
	Moderate and severe addiction level (*N* = 58)	52.86	10.737	.72	.998	.83	.965	.19	.951
*TOTAL (N = 153)*		35,16	16.913	.02	.979	.20	.973	−.11	.879
*TOTAL*	No addiction (*N* = 86)	11.90	4.746	−.70	.355	−.88	.498	−.52	.529
	Low level of addiction (*N* = 136)	29.78	5.603	−.21	.699	−.08	.775	−.06	.901
	Moderate and severe addiction level (*N* = 126)	52.53	1.796	.70	1.130	.69	.967	.41	1.162
*TOTAL (N = 348)*		33,73	17.640	.00	1.002	.01	1.000	.01	1.000

**Table 4:** The results of multivariate analysis of variance with gender and IAT categories as independent variables and IAT scores and factors as dependents variables

Independent variable	Dependent variable	Multivariate test η_p_^2^	Univariate test η_p_^2^
Gender	IAT score	.062**	.004
	Em. and cog. Preocc.		.001
	Neglecting work		.029**
	Social problems		.014*
IAT categories	IAT score	.410**	.801**
	Em. and cog. Preocc.		.916**
	Neglecting work		.352**
	Social problems		.124**
Gender*IAT Categories	IAT score	.009	.006
	Em. and cog. Preocc.		.001
	Neglecting work		.002
	Social problems		.006

* *p* < .05

** *p* < .01

The results of multivariate tests showed that there are significant differences regarding gender (F = 5.619; *p* < .01) and IAT components (F = 58.958; p < .01), while the gender and IAT category interaction was not significant (F = .780; *p* > .05). With regard to the way of forming the categories the differences on IAT by IAT categories have low scientific importance. However, it is interesting to notice the difference in the size of the effects where the effect of the differences for the *Emotional and cognitive preoccupation with the internet* is extremely high, while for the other two factors is relatively low. It can be concluded that the highest importance of the role in internet addiction risk goes to *Emotional and cognitive preoccupation with the internet*, while *Neglecting work and lack of self-control* and *Social problems* are of lesser importance.

**Fig. 1:**
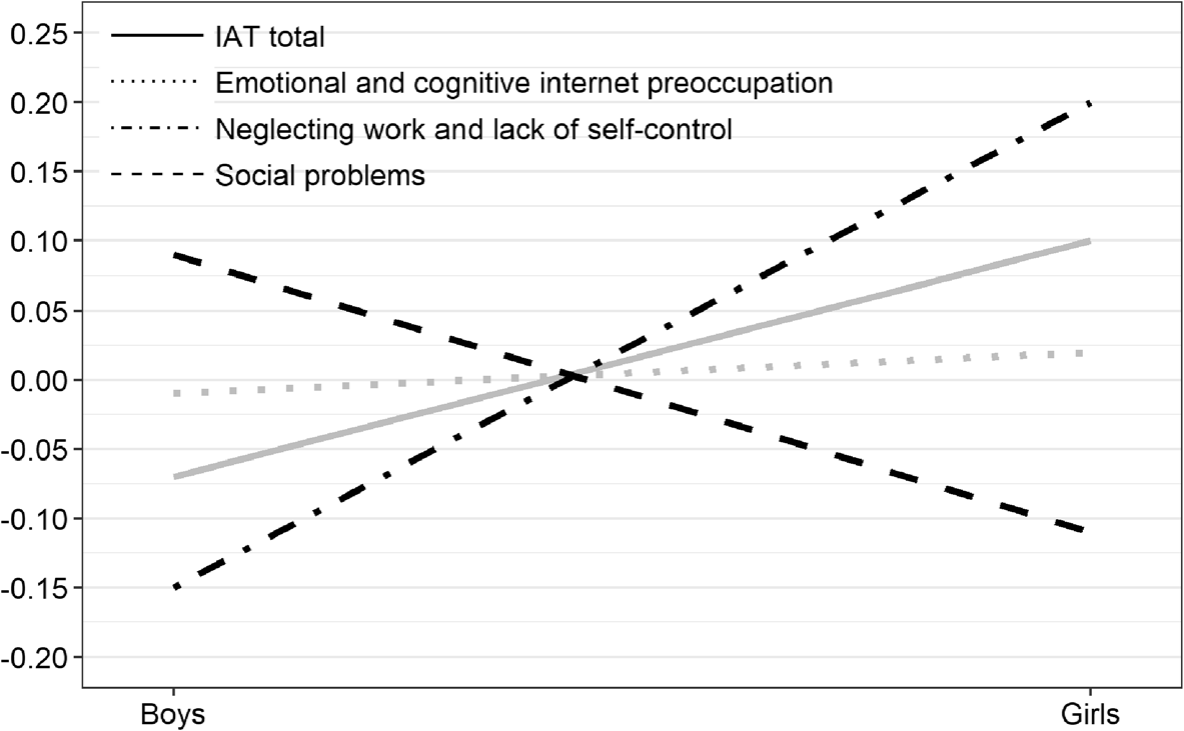
Gender Differences on Total IAT Score and Factors as Z values

By examining gender differences, a statistically significant multivariate effect is shown, which is why univariate effects have been explored. There have been differences in Neglect of work and a lack of self-control and Social problems, while significant differences in Emotional and cognitive preoccupation with the internet and the overall score have not been found. Figure 1 shows the results of gender differences. In order to allow a visual comparison, the overall results on the IAT test are standardized to z-values.

More detailed analysis of significant gender differences on two factors shows a different pattern for boys and girls. Thus, girls achieve significantly higher results than men on the factor *Neglect of work and the lack of self-control*, while the *Social problems* factor shows significantly lower results for girls than for boys.

## Discussion

The aim of this study was to determine the level of internet addiction on a sample of high school students in Croatia. Since this is the first significant, preliminary representative research of Internet addiction on high school population in Croatia, the results of prevalence of Internet addiction on this sample are also significant for the national coverage. At the same time, the purpose of the research was to provide national results from the research conducted by standardized instrument to allow comparable data with internationally available findings. In order to determine the level of addiction to the internet on individual dimensions, it was necessary to undertake the factorization of the IAT. Specifically, previous studies have shown that, despite the high internal homogeneity of the test, there are numerous variations in the factor structure [8, 19, 22, 30, 50]. On the sample of high school students in Croatia, a high internal consistency of the whole test was determined (.91). Principal components analysis found that the IAT on a high school sample has a three-factor structure with dimensions:

- Emotional and cognitive internet preoccupation - reliance on online life
- Neglecting the work and lack of self-control,
- Social problems

Although many structural variations have been identified in the research, some similarities with previous research exist. Chang and Law [8], and Lai et al. [31] in their research conducted in China also gained a three-factor structure of the test with similar dimensions as we did. Their dimensions of withdrawal and social problems point to a high degree of correspondence with the dimension of “social problems” obtained through this research, while the definition of time management and performance points to similarities with neglect of work and lack of self-control. However, the third dimension obtained in both previous studies on a sample of Chinese students - the substitution of reality, although similar in content, points to deviations from the dimension of *Emotional and cognitive internet preoccupation*, given that, in the study presented here, this dimension has a wider range and contains a larger number of items. Although the greatest resemblance to this research exists with the studies conducted in China, we find some similarities with studies conducted in European countries of a more similar cultural environment. Dimensions similar to *Emotional and cognitive internet preoccupation* were obtained in German [3] and the Finnish sample [42], and similarities with the dimension *Neglect of work and lack of self-control* were also obtained in German [3], Italian [19] and the English sample [50]. The dimension of *Social Problems* points to the equivalence to the dimension obtained in Italian [19] and the English sample [50].

In order to examine the level of internet addiction on a sample of high school students in Croatia, the students were categorized into four proposed categories by authors and researchers [50, 53]. The highest number of high school students belongs to the low-level addiction category (39%). This score represents the average online users with complete internet usage control. Only 3.4% of high school students belong to the category with a severe level of addiction, but up to 32% of the students belong to the moderate level of addiction. According to the author’s instructions, the score of more than 40 on the test indicates that respondents show signs of addiction on the internet. The category of respondents belonging to the moderate level of addiction level has a score of more than 40 and can be considered a sign of addiction to the internet. We can conclude that 35.4% of high school students report signs of internet addiction suggesting the risk of more serious problems in using the internet. In a recent meta-analysis of 164 independent samples of 31 countries, the highest prevalence of high internet addiction was found in the Middle East (10.9%). The following are Southeast Europe with a prevalence of 6.1% while the North and West Europe countries show the lowest percentage of prevalence of internet addiction (2.6%). In the sample of high school students, this percentage is 3.4% and is somewhat higher than the average of Western European countries. Nevertheless, research conducted in Italy on a large sample of young people aged 14 to 21 shows that less than 1% of young people show a high level, while only 5% show a moderate level of addiction. This percentage is far less than 35.4% of students in Croatia who belong to these two categories. Based on the results on IAT dimensions in our sample it is evident that pupils who show signs of internet addiction achieve the highest results on the dimension of *Emotional and cognitive internet preoccupation*. Based on the multivariate tests conducted in this study, it is possible to conclude that the majority of the role in internet addiction risk has *Emotional and cognitive internet preoccupation*, while *Neglecting work and lack of self-control* and *Social problems* are of lesser importance. Similar results were obtained from the authors of the study conducted on a sample of students in Finland [42], where the intergroup difference on the dimension scores among groups based on the addiction risk is the largest in the similar dimension - reliance on online life.

Gender differences in the level of internet addiction were also the subject of this research. Univariate testing did not confirm the existence of gender differences in the overall outcome of the test, but multivariate testing on dimensions and overall results confirmed the existence of gender differences. Girls achieve higher results than boys on *Neglecting work and lack of self-control* dimension, while at the *Social Problems* factor girls show statistically significantly lower results than boys. It is interesting to note that on the dimension *Emotional and cognitive internet preoccupation* that plays the main role in the internet gender differences were not confirmed. The results obtained are in line with some of the previous research. Lai et al. [31] also confirm that greater social problems due to online addiction are reported by men. The lower score of female respondents on the dimension of *Neglecting work and lack of self-control* is consistent with the results of Sinkkonen, Puhakka & Meriläinen [42] that confirm that female respondents report less self-control when using the internet than male respondents. These results indicate that gender differences on internet dependency cannot be inferred in general, on the overall outcome of the test, but it is also necessary to examine specific differences in some aspects of internet addiction. Gender differences should also be considered in the context of time spent on the internet. The greater risk of internet addiction is probably linked to different patterns of internet usage. Men spend more time playing games online, while women spend more time on social networks and e-mails, and the activity of playing games shows a risk towards addictive behavior [17]. Therefore, different ways of expressing addiction for men and women can be related to different forms of internet usage. In further research, it is also necessary to examine ways of using the internet in high school samples.

The results of this study should be considered in the context of some disadvantages and recommendations for further research. One of the main disadvantages of this study was the clusterization of the sample on the grade division level. While due to the ethical considerations we were unable to collect the data on the specific grade division of the participants and control for the grouping effects, it is recommended for future studies to examine clusterization effects using multilevel models. In addition to examining the usage of the internet, it is also necessary to examine the time spent on the internet that has not been investigated by this research. The question is whether individuals are dependent on the internet in general, or on certain activities on the internet. Earlier research suggests that interactive internet functions are associated with its excessive use and that individuals involved in online interactive applications show a tendency to addictive behaviors ([15, 33]; Li & Chung, 2006). This study did not investigate how much time the respondents spent on certain activities on the internet, and did not distinguish the activities on the internet for educational purposes and leisure activities. Some shortcomings of this research are reflected in the very shortcomings of IAT as the questionnaire that was used. The item that has the smallest correlation with the overall score on the test in this research is about how often students ignore intimacy with their partner. Finding of this preliminary research propose that this assessment on intimacy with the partner is presumably not appropriate for high school students and this should be taken into account in future research. Adaptation of internet addiction measures for different age groups is necessary in order to be applicable in all age groups. Also, given that the structure of the test itself has high variations between cultures and also between the countries of similar cultures, the question whether the diagnostic criteria of test results and categorization of subjects should be the same in all test applications. It is therefore necessary to measure cultural invariance of IAT and to conduct validation studies using contrast groups of children who exhibit the symptoms of internet addiction in clinical situations and children who do not exhibit such symptoms.

In the future, it is necessary to examine the temporal stability of this construct. Temporal stability and repeated need for use of addictive substances are the criteria for assessing all forms of addiction (ICD-10). Lam [32] used the IAT to test its temporal validity and found that only 17% of subjects who showed signs of addiction had the same results re-applied after 2 years. The question is whether these findings are the result of the internet addiction itself or the fact that internet addiction is not a mental disorder. In the upcoming version of this ICD-11 classification (expected in 2018), it still discussed whether the problematic use of the internet is an independent disorder or the internet is a medium where various forms of dysfunction of the impulse control disorder are manifested. Studies on the temporal stability of this construct are necessary to determine if internet addiction has the diagnostic prerequisites to be classified as the independent mental disorder. On the other hand, in the context of the DSM-5 manual ([2], pp. 795), the possibility of the inclusion of the internet Gaming Disorder in the further editions of the manual is being discussed. Internet addiction is not mentioned here because of the ambiguity of discerning non-normative behavior in the context of business obligations or other disorders (eg. sexual content) and in the DSM it is stated that additional research is needed to determine internet usage patterns that correspond to the diagnostic criteria of addiction (pp. 798). So far, only gambling addiction has been included among addictions even though there is no active substance, but research using IAT points to subjective problems indicated by the use of the internet. These measures, though expressed as a self-assessment of the condition, point to practical significance in dealing with adolescents, especially in situations where daily functioning is impaired and may be an indicator of some other difficulties. It is also important to systematically monitor the prevalence of internet addiction indicators as well as the difficulties associated with excessive use of the internet to provide a measure of difficulty detection and comorbidity with other disorders. Such a measure is especially needed for monitoring the impact of prevention and intervention programs.

## Conclusion

To conclude, this application of the Internet Addiction Test to the sample of high school students in Croatia found that 3.4% of high school students reported high levels of internet addiction, while 35.4% of respondents reported some signs of addiction. Cognitive and emotional preoccupation with the internet has the most significant role in internet addiction risk. This finding can be used by practitioners in developing interventions as well as prevention of internet addiction. The prevention programs should be focused on the constructive use of adolescents’ own free time. Prevention activities, as well as treatment of young people with a high level of internet dependence, need to focus on the area of emotional and social competence as well as the responsible use of media content and modern technology. The analysis of gender differences showed that although there are no significant effects on the total score, there are differences on *Social problems* where boys score higher than girls and on *Neglecting work and lack of self-control* where girls score higher than boys. Program interventions on safe and adequate ways of using the internet are necessary at an earlier age, in order to reduce the risk of high levels of internet addiction in the adolescent period. A group that needs special attention to prevention is the one that shows signs of moderate levels of addiction on the internet. Given that this category is the third of our sample, it is clear that programs of both indicated and selective prevention should be systematically planned for the general population of adolescents as well as for the groups in risk. Further studies in this area need to be set up within the framework of a systematic, multivariate, psycho-social conceptual framework, and it would be interesting to check out the relationship between the level of addiction to the internet and the emergence of other problematic behaviors of young people such as the substance abuse and other behavioral problems present in the population of young people.

## Data Availability

The datasets used and analyzed during the current study are available from the corresponding author on reasonable request.

## Abbreviations

DSM: Diagnostic and Statistical Manual of Mental Disorders
IA: Internet addiction
IADQ: Internet Addiction Diagnostic Questionnaire
IAT: Internet Addiction Test
ICD: International Classification of Diseases and Related Health Problems
I-PACE: Interaction of Person-Affect-Cognition-Execution model
KMO: Kaiser Meyer Olkin coefficient
MANOVA: Multivariate analysis of variance
WHO: World Health Organization
